# A New Environmentally Friendly Mortar from Cement, Waste Marble and Nano Iron Slag as Radiation Shielding

**DOI:** 10.3390/ma16072541

**Published:** 2023-03-23

**Authors:** Ahmed M. El-Khatib, Mahmoud I. Abbas, Mohamed Abd Elzaher, M. Anas, Mohamed S. Abd El Moniem, Mahmoud Montasar, Ebeid Ellithy, Mahmoud T. Alabsy

**Affiliations:** 1 Physics Department, Faculty of Science, Alexandria University, Alexandria 21511, Egypt; 2 Department of Basic and Applied Science, Faculty of Engineering, Arab Academy for Science, Technology and Maritime Transport, Al Alamein P.O. 1129, Egypt

**Keywords:** cement mortars, waste marble, nano iron slag, linear attenuation coefficient, half value layer, shielding parameters

## Abstract

Improving mortar shielding properties to preserve environmental and human safety in radiation facilities is essential. Conventional cement mortars, composed of cement, water, and lime aggregate, are crucial for radiation shielding. Using recycled aggregates to produce new mortar and concrete compositions has attracted the attention of several researchers. In the current study, waste marble and iron slag as aggregates are used to create novel cement mortar compositions to study the aggregate’s impact on the radiation attenuation capability of the mortar. Three mortar groups, including a control mortar (CM-Ctrl), were prepared based on cement and waste marble. The other two groups (CM-MIS, CM-NIS), contained 25% iron slag at different particle sizes as a replacement for a waste marble. The study aims to compare iron slag in their micro and nano sizes to discuss the effect of particle size on the mortar radiation capability. For this purpose, the NaI scintillation detector and radioactive point sources (^241^Am, ^133^Ba, ^137^Cs, ^60^Co, and ^152^Eu) were utilized to measure several shielding parameters, such as the linear attenuation coefficient (LAC), mass attenuation coefficient (MAC), half-value layer (HVL), tenth-value layer (TVL), and mean free path (MFP), for the produced mortars at different photon energies. Furthermore, the transmission electron microscope (TEM) is used to measure the particle size of the aggregates. In addition, a scanning electron microscope (SEM) is utilized to acquire the cross-section morphologies of the prepared mortars. According to our findings, mortars prepared with nano-iron slag and waste marble offered superior shielding capabilities than mortars containing natural sand or fine crushed stone. The nano iron slag mortar can be utilized in place of typical sand mortar for applications as rendering or plastering materials for building medical diagnostic and CT scanner rooms, due to its improved shielding abilities.

## 1. Introduction

Hundreds of uses for ionizing radiation exist today in a variety of industries, including energy production and medicine. Despite the advantages of ionizing radiation, care must be taken when working with radioactive sources because high-energy photons can be quite hazardous to the human body [1]. As a general rule, people should spend as little time as possible in close proximity to radiation sources and keep as far away as feasible. Shielding offers an efficient way to safeguard staff and patients; this is not always practicable, and often further precautions are required. Depending on the application, various materials are frequently employed for this function. For example, concrete is commonly used as the absorber to line the walls of X-ray rooms, due to its practicality and capacity to attenuate X-rays effectively. Even though concrete is often an excellent choice, different materials are occasionally required, since concrete is prone to cracking and loses water after prolonged radiation exposure [2]. High-density materials and heavy elements like lead and barium are the best at absorbing radiation doses [3]. Lead and lead compounds have been used to shield against the dangerous effects of ionizing radiation [4]. Lead has typically been employed in clinical settings for technician and patient safety in the field of X-ray, nuclear medicine, and equipment containers, due to the low cost and potential for radiation shielding. Unfortunately, despite its advantages, lead is poisonous [5] and has poor mechanical qualities. The shielding efficiency of several materials and composites have been studied, and their linear attenuation coefficients (LAC) were determined [6,7]. The novel substance offers a safe, economical replacement for conventional materials that are toxic and hazardous to the environment. The creation of innovative materials in current research has focused chiefly on composites consisting of high-density metals and various construction components [8].

Mortar is a material used to cover internal building walls. It simply consists of cement and sand. Cement mortars have long been a popular solution for nuclear radiation shielding. Mortar is being utilized more frequently as a protective material in applications that use radiation to filter out incoming photons that could harm patients and staff near the radioactive source. It has a high gamma attenuation coefficient and can be cast in various configurations. Several aggregates or additions have been tested on concrete, and numerous studies have been conducted to improve the gamma-ray shielding capacity of cement mortars [9]. The shielding properties of mortar could be improved by using materials of higher density than sand, where the density plays an important role in enhancing the shielding properties. For instance, barite is one of the most-studied cement blends in this context; hence, it has better attenuation features than other mortar systems [10].

Growing attention is being focused on the need for sustainable materials. It is preferable to use paper, plastic, glass, and metal wastes as raw materials rather than burying them in disposal locations. Utilizing recycled materials reduces the amount of waste sent to landfills while lowering raw material extraction and pollution [11]. The opportunity of using recycled aggregates to produce new mortar and concrete compositions has attracted the attention of several researchers. Different types of recycled aggregates, such as waste glass [12,13], rubber tree seed shells [14], coconut fiber [15], bottom ash [16], PET plastic waste [17], waste marble [18,19], and iron slag [20], have been investigated as replacements for aggregate.

Marble is utilized in various applications, including walls, flooring, décor, household objects, and antiquities. Manufacturers generate massive amounts of marble waste in the form of powder and irregular stones, polluting the agricultural and animal environments. This marble debris can be used instead of traditional natural coarse aggregate in concrete [21]. Recently, Alabsy et al. developed a radiation-protective mortar based on gypsum, lime, and waste marble and loaded with lead oxides of different particle sizes [22]. Iron slag is a byproduct of steel manufacturing that contains substances such as recycled steel scrap, coke, lime, and metal oxides [23]. Around one ton of slag waste is generated for every three to four tonnes of stainless steel produced [24]. Slags are employed in a variety of applications daily, including Portland cement production [25], agricultural fertilizer [26], and mineral CO_2_ sequestration [27]. Furthermore, iron slag has been examined as an aggregate replacement to improve γ-ray shielding properties of concrete [28].

The MAC is experimentally determined for a specific medium to determine how effective a shield is. The general shielding capacity of a medium is described by its MAC, which also accounts for density. Because additional parameters are calculated using this parameter, the MAC value’s precision must be exact. To achieve this, it is common to compare the MAC values to the theoretical values of the prepared materials and then assess the % difference. Other shielding parameters, like the half-value layer (HVL), tenth-value layer, and mean free path (MFP), are evaluated once the MAC results are considered valid [29].

This study’s main aim is to employ waste products from the marble and steel industry to advance the industrial sector’s environmental and commercial interests while improving the effectiveness of radiation shielding using affordable, locally available waste materials. For this purpose, the shielding characteristics of mortar samples made from cement-waste marble-iron slag were examined. Cement, iron ore, and concrete are commonly used worldwide in the production of shielding materials. However, nano iron slag is not common in producing these materials. The experimental efforts intended to explore the effect of particle size (either micro or nano) of iron slag incorporated in cement-waste marble mortar samples on attenuating gamma rays. The radiation shielding ability for micro and nano slag was experimentally measured between 0.0595 and 1.41 MeV. The importance of the current study is to develop the walls of radiation facilities with low-cost mortars friendly to the environment and capable of absorbing gamma or X-rays more efficiently.

## 2. Materials and Methods

### 2.1. Materials

**Figure 1:**
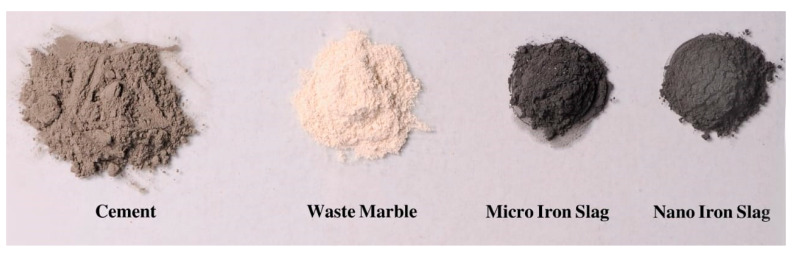
The utilized materials in powder form after sieving.

**Figure 2:**
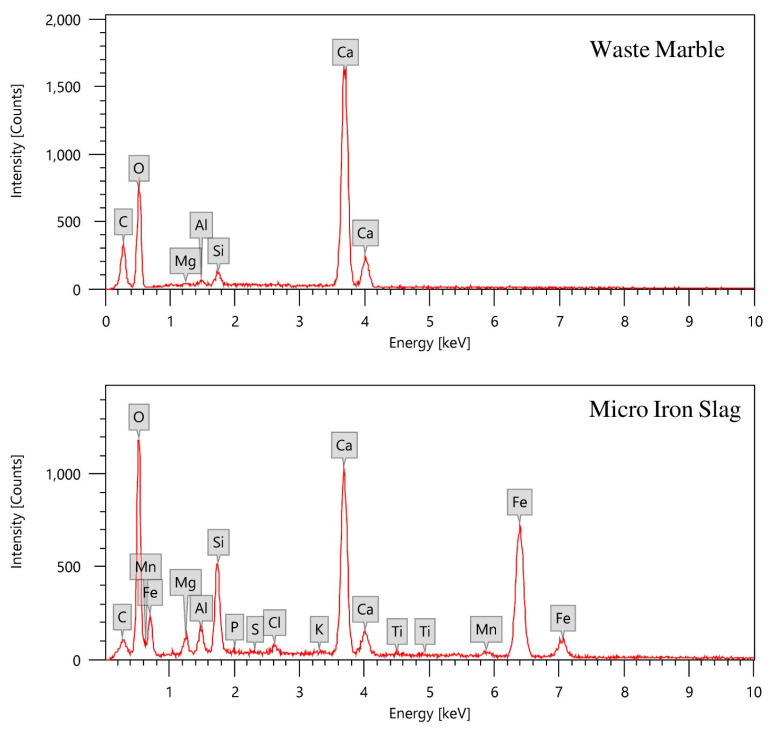
EDX spectra of waste marble and micro iron slag.

Materials used in this investigation included Portland cement (supplied locally) and powdered micro iron slag, which is a by-product created during the steel production process, and was provided by Ezz Steel Company in Egypt. Additionally, powdered waste marble collected from marble factories was dried, ground by a mechanical grinder, and then sieved to be used as an aggregate. The nano iron slag powder was prepared by ball milling as described in Section 2.2. The utilized materials in powder form after sieving are shown in Figure 1. Table 1 lists the elemental analysis of cement, waste marble, and iron slag by employing energy-dispersive X-ray spectroscopy (EDX) analysis. The EDX spectra of waste marble and iron slag powders are depicted in Figure 2. Moreover, water was added in the mixing process to prepare the mortar.

### 2.2. Synthesis of Nano Iron Slag

Nano iron slag powder was produced by high-energy planetary ball milling (Fritsch Pulverisette 7, Fritsch, Weimar, Germany) at a rate of 500 rounds per minute (rpm). The ball mill contains four vials of size 50 mL made from tungsten carbide. Balls of different sizes with a total mass of 90 g and a diameter between 2 and 10 mm were employed in the milling process, where the ball-to-powder weight ratio was set to be 5:1.

### 2.3. Mortar Sample Preparation

**Figure 3:**
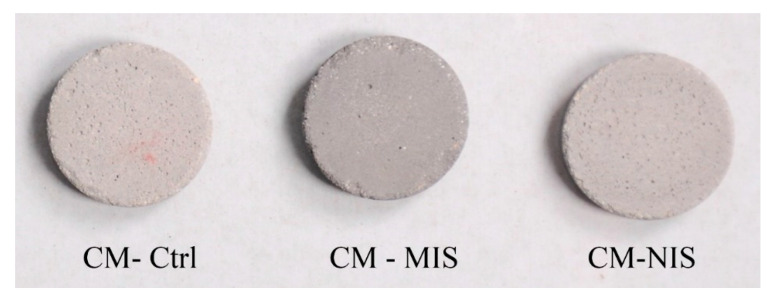
The prepared mortar samples.

The mortar was made by mixing cement with waste marble and then adding water in a 1:0.5 cement-to-water ratio and agitating well to obtain a homogenous mortar of control sample (CM-Ctrl), and then adding iron slag with different particle sizes (micro and nano) to obtain the rest of the mortar samples (CM-MIS, CM-NIS). Table 2 lists the codes of the mortar groups along with the mixing ratios. All the mortar samples were poured into cylindrical rubber molds with dimensions of 30 mm in diameter and 5 mm in height. The prepared mortar samples are shown in Figure 3. Each group contained three specimens. All specimens were placed in a sunny place for more than a week to ensure consistency and hardness. The density of each mortar sample was conventionally measured by applying the Archimedes principle. To achieve this, a calibrated single pan electrical balance with accuracy 0.0001 g was used to weigh the samples and experimentally estimate volumes for the cylindrical samples.

### 2.4. Radiation Measurement Setup

**Figure 4:**
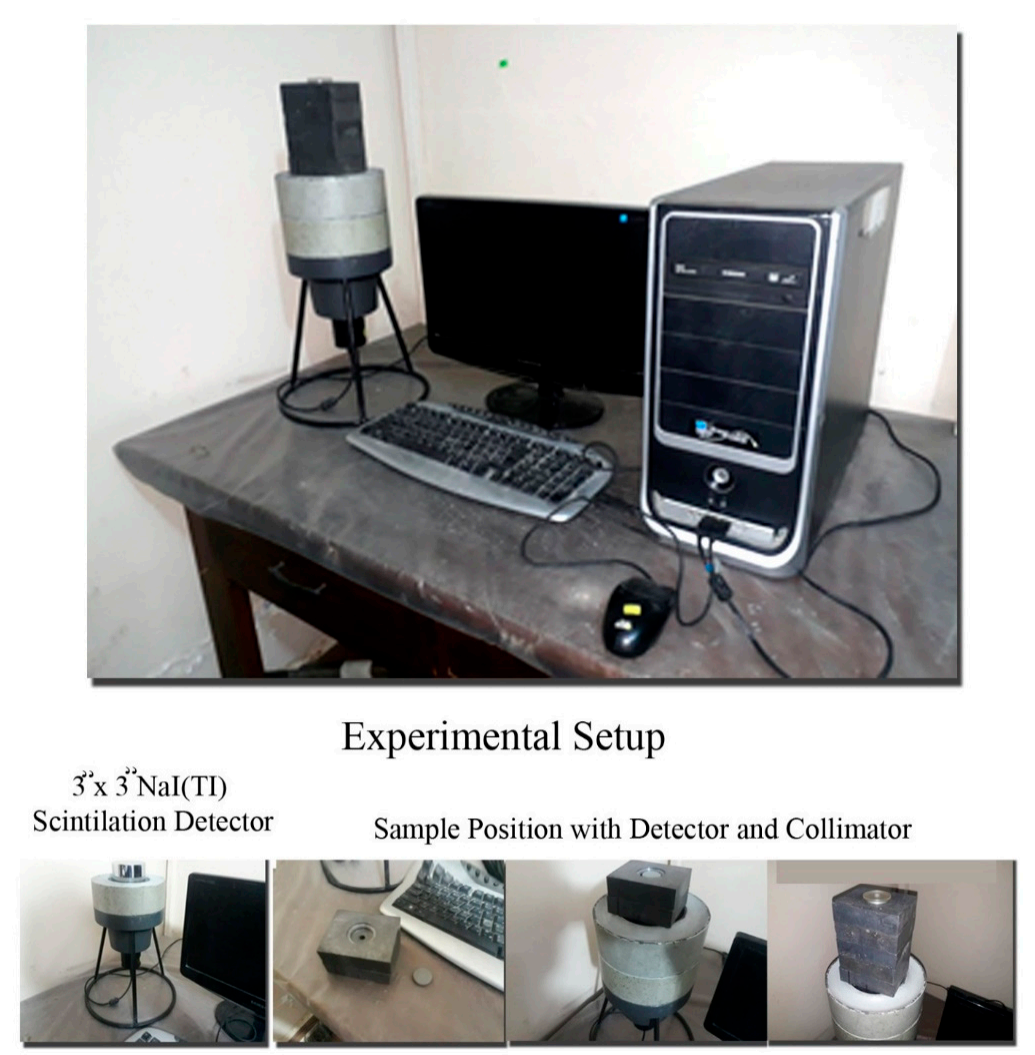
The experimental setup arrangement for gamma-ray measurements.

The experimental gamma ray measurements were performed by a well-calibrated [30] “3 × 3” NaI (Tl) Gamma spectrometer as displayed in Figure 4. Five standard radioactive point sources in the energy range 59.53 keV to 1408.01 keV are displayed in Table 3. The emerging photons from the examined mortar interacted with the detector, which converted them into electrical signals with different sizes and displayed them as peaks in a spectrum via the Genie 2000 software. The gamma spectra for all the measurements were recorded after a sufficient number of times such that the statistical error would be less than 1%. Then, for every energy and thickness, the net area under each peak in the spectrum was inserted in an excel sheet to compute the shielding parameters of the investigated composites. The experimental values of attenuation coefficients were compared to those from the XCOM program to verify the validity of the experiments.

### 2.5. Theoretical Approach

The linear attenuation coefficient (*μ*) or LAC (cm^−1^) is computed empirically using the well-known Beer-Lambert’s law and is defined as the probability of photons interacting with matter per unit length and is given by Equation (1) [31]:

(1) $$\mu =\frac{1}{x}ln\left(\frac{I_{0}}{I}\right)$$

where $I_{0}$ and I are the incident and transmitted intensities, respectively, passing through a target material of thickness *x*.

Notice that the mass attenuation coefficient or *MAC* (*μ*/*ρ*) can be established by dividing the experimental linear attenuation coefficient (*μ*) of sample by its density (*ρ*) as given in Equation (2) [32],

(2) $$MAC=\frac{\mu }{\rho }$$

To confirm the validity of the experimental data, *MAC*s were calculated theoretically by using NIST XCOM online program [33]. It is worthwhile to extend the calculations by using the values of LACs to calculate the other shielding parameters of the investigated mortars *HVL*, *TVL*, and *MFP*. The *HVL* and *TVL* are defined as the thicknesses required to attenuate the incident photon intensity by factors of 1/2 and 1/10, respectively, and are calculated using the following relations [34,35]:

(3) $$HVL=\frac{\ln2}{\mu }$$

(4) $$TVL=\frac{\ln10}{\mu }$$

The *MFP* (cm) is defined as the average distance that a photon travels inside the sample without any interactions [36]:

(5) $$MFP=\frac{1}{\mu }$$

## 3. Results and Discussion

### 3.1. Microstructural Characterization

#### 3.1.1. Transmission Electron Microscope (TEM)

**Figure 5:**
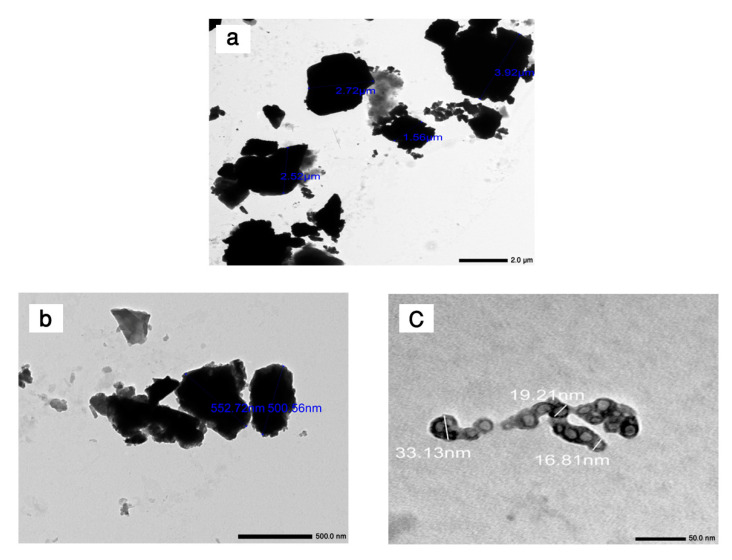
TEM images of (**a**) waste marble, (**b**) micro iron slag, and (**c**) nano iron slag powders.

The size of the aggregates was analyzed by employing the TEM (JEM2100F, JEOL, Tokyo, Japan) at 200 kV. The sample was prepared by dispersing the powder in ethanol by ultrasonic vibration on a Cu grid. The TEM micrographs of waste marble, micro iron slag, and nano iron slag particles are displayed in Figure 5. It is clear from Figure 5a,b that waste marble powder and micro iron slag particles have irregular and nonhomogeneous shapes with an average particle size in the order of 2.75 µm and 0.5 µm, respectively. On the other hand, Figure 5c reveals that nano iron slag had a uniform shape with particle sizes ranging from 16 nm to 33 nm.

#### 3.1.2. Scanning Electron Microscope (SEM)

**Figure 6:**
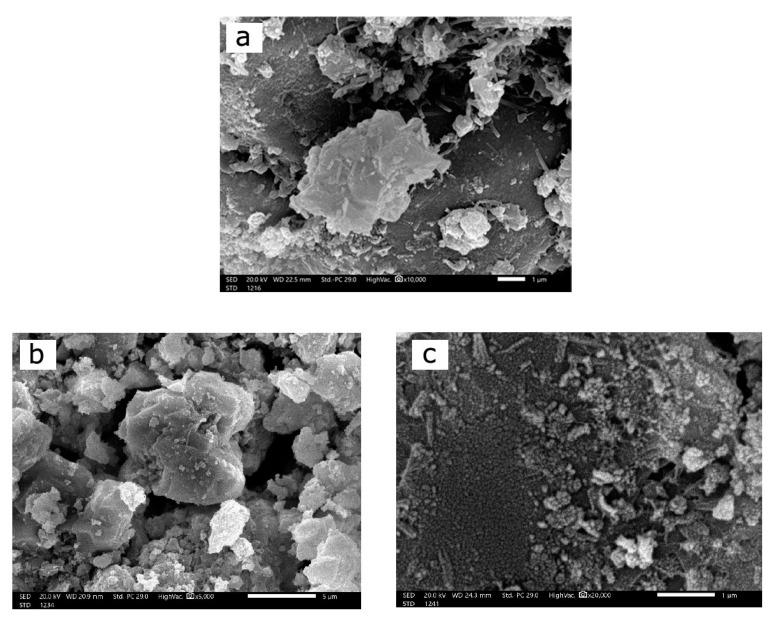
SEM images of (**a**) CM-Ctrl, (**b**) CM-MIS, and (**c**) CM-NIS mortar samples.

The prepared mortars’ cross-section morphologies were investigated using SEM (JSM-6010LV, JEOL) to examine the distribution of the reinforced materials in the mortar mixtures, as depicted in Figure 6. Before the SEM examination, the mortar samples were covered with a fine layer of gold under vacuum using an ion-sputtering coating device (JEOL-JFC-1100E). It is evident from the SEM images in Figure 6a–c that the morphologies of the CM-Ctrl, CM-MIS, and CM-NIS mortar samples differed significantly, and there were discrepancies in the dispersion of the micro and nano slag within prepared mortars. The SEM image of the CM-Ctrl sample in Figure 6a displays the morphology of the control mortar containing voids. Adding micro slag powder as aggregate replacement in the CM-MIS sample improved the mortar structure, as seen in Figure 6b. On the other hand, Figure 6c reveals that the distribution of iron slag nanoparticles in the CM-NIS sample was more uniform and homogenous than that of the micro iron slag. Due to their tiny size and properties, iron slag nanoparticles efficiently raised the homogeneity within the mortar, decreased the ratio of gaps in the sample, and consequently improved the gamma-radiation shielding properties.

### 3.2. Shielding Parameters

**Figure 7:**
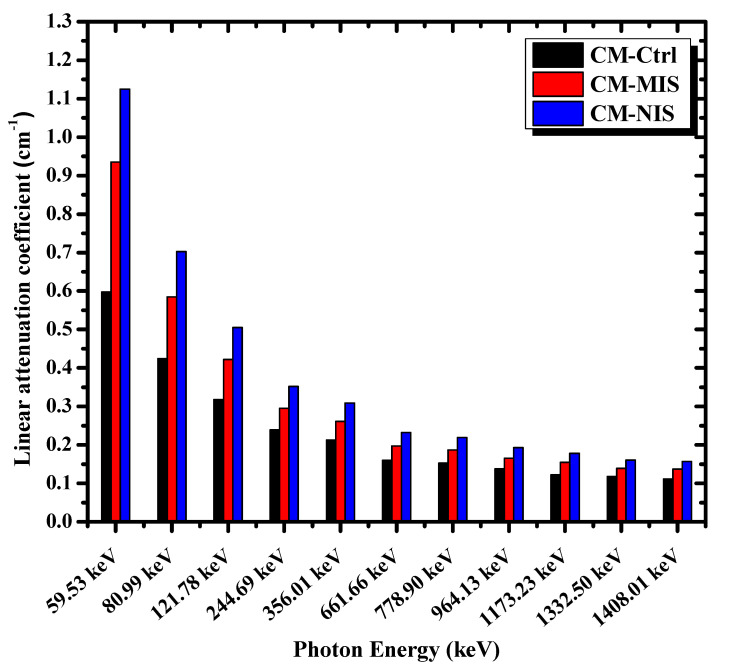
The histogram of the linear attenuation coefficients of the tested mortars as a function of photon energy.

Using the experimental setup, it was easy to determine the values of LACs of the tested mortars. These values were tabulated in Table 4 and displayed in Figure 7. It is worthy of notice that the LACs for all the studied mortars decrease with increasing photon energy. This tendency is the usual behavior for photons inside any attenuating materials, except those with K-edges. Of course, the energy of the photon and the type of absorber, besides its morphology, play an essential role in determining precisely the type of interaction mechanisms. At low energy, the predominant interaction of photons is the photoelectric effect, while for photon energy greater than 200 keV, Compton scattering is dominant up to 2 MeV. Greater than these energy values, Compton scattering will compete with the pair production interaction. At higher values, the pair production interaction will be predominant [37]. To explain the superiority of these new mortars, a comparison was made between the LACs of CM-Ctrl, CM-MIS, and CM-NIS mortars and the values of LACs for ordinary mortar (cement and sand) obtained from reference [7], as mentioned in Table 4. According to Table 4, the values of LACs of the three prepared mortars are greater than those of ordinary mortars at any photon energy, even those of the control mortar CM-Ctrl.

However, iron slag in both micro and nano forms as reinforced materials highly improved the shielding properties of the mortar at any photon energy used in this work, as presented in Table 4. This is attributed to the shape and distribution of the reinforced particles inside the mortar. This distribution, plus the formation of the aggregates, highly affects the photon’s mean free path at a particular photon energy. Moreover, the mortar CM-NIS has the highest values, which is also clear from the calculated LAC ratios in Table 5; it is considered the best of the three for use as a shield against photons, especially in X-ray rooms.

To confirm the high validity of the experimental results, the mass attenuation coefficients (MACs) were calculated experimentally by using Equation (2) and theoretically by the XCOM database [38]. The chemical compositions of each reinforced mortar (CM-Ctrl and CM-MIS) were entered into the XCOM program to get the theoretical data. The obtained calculations from the experimental MACs (Equation (2)) and the XCOM program were tabulated in Table 6. The experimental results were in good agreement with those calculated by the XCOM software. The deviations between their values are not greater than 2.5%, as given in Table 6.

Other essential shielding parameters, HVL, TVL, and MFP, were calculated using Equations (3)–(5), respectively. Again, the nanocomposite significantly affects the attenuation coefficients, and the same performance was observed for HVL, TVL, and MFP, as shown in Table 7, where the values for lead were added for comparison. As provided in Table 8, one can determine how much thickness is required to cut radiation exposure in half. For energies above 700 keV, we may use the thickness of CM-NIS mortar fourfold as the lead.

**Figure 8:**
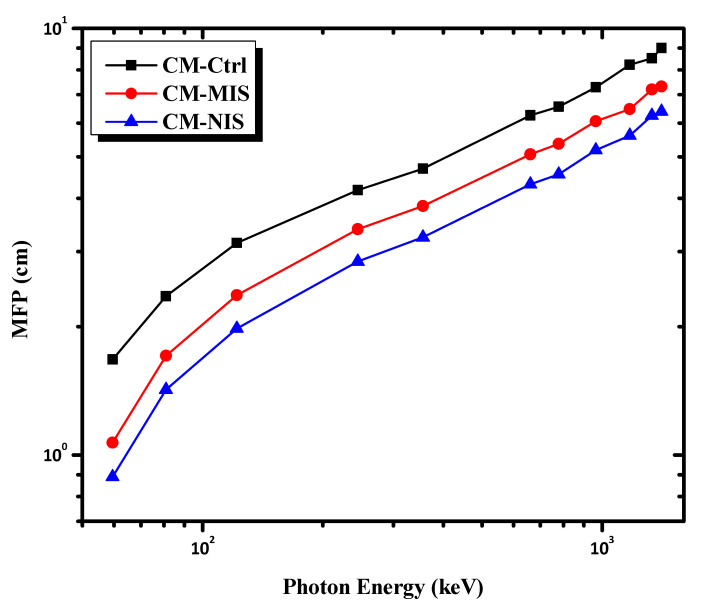
The variation of the mean free path as a function of photon energy.

Figure 8 displays the MFP for each sample. Within the range, the lowest MFP value is found at the lowest tested energy, 59.5 keV, and the highest MFP is obtained at 1408 keV. This upward behavior happens because higher energy radiation can penetrate the incident material easily. Compared to the Compton interaction, the photoelectric effect becomes less dominant at higher energies [39]. A denser material will have more interactions between photons and atoms, resulting in more significant attenuation. In other words, the density of a material influences the likelihood that radiation entering the shield will interact with it. At all energies, the mortar reinforced by nano-iron slag has the lowest MFP, which means more photon interactions. For instance, CM-NIS has MFP values 0.889 and 6.386 cm, at energies 59.5 keV and 1408 keV, respectively, compared with those for CM-MIS mortar which has values of 1.069 and 7.310 cm at the same energies. This explains how the atomic distribution and the surface area in nano-size samples play an important role in determining the number of photon interactions.

**Figure 9:**
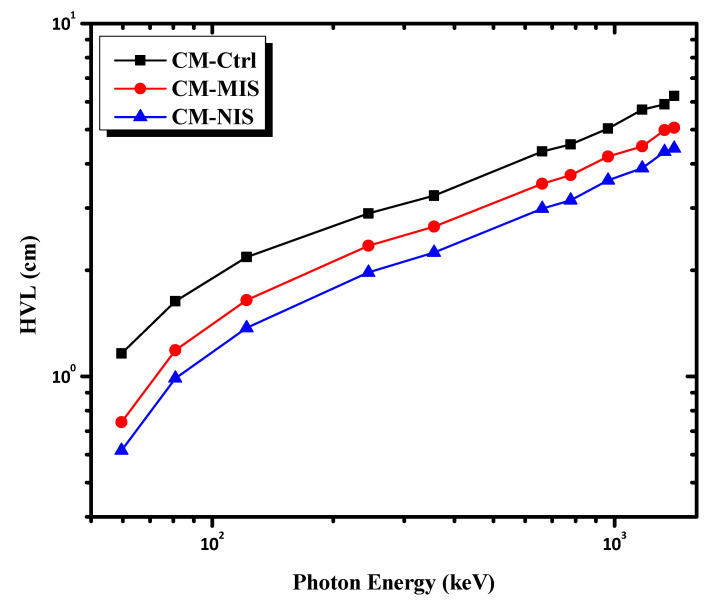
The variation of the HVL as a function of photon energy.

**Figure 10:**
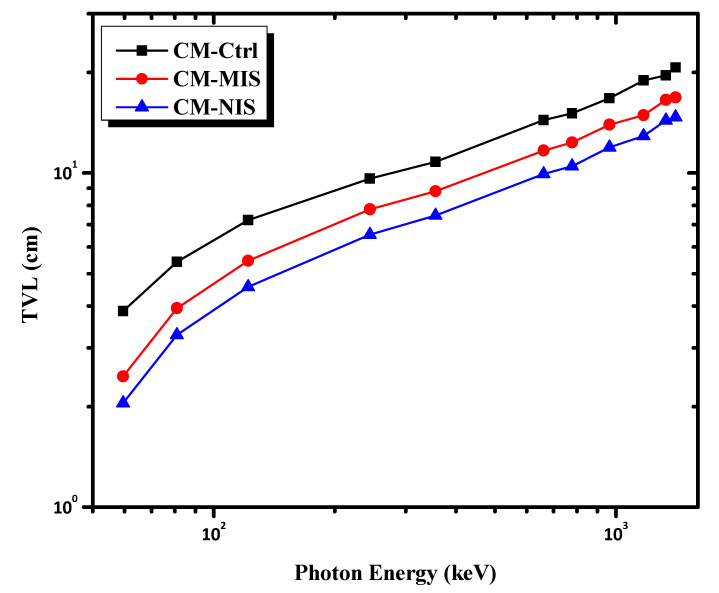
The variation of the TVL as a function of photon energy.

A frequent parameter used in research on radiation shielding is the HVL. The lower the HVL of a material, the higher the radiation shielding. Figure 9 shows the HVL as a function of the incident photon energy for the investigated mortars. As can be seen from Figure 9, the HVL values increase in the order of CM-NIS < CM-MIS < CM-Ctrl. The mortar loaded with nano iron slag has the lowest HVL and the best shielding ability. In contrast, the control sample has the highest HVL and, consequently, the lowest shielding capability. Furthermore, Figure 9 indicates that the HVL for all the investigated mortars increases with increasing photon energy. The HVL of the CM-NIS mortar rose from 1.970 to 2.989, 3.597, 4.329, and 4.426 cm for energies of 244, 661, 964, 1332, and 1408 keV, respectively. In Figure 10, the TVL is also studied for all samples within the energy range. It showed the same behavior as HVL in the former analysis.

## 4. Conclusions

In this research, innovative cement-mortar compositions based on waste marble and iron slag as aggregates were prepared to examine the radiation attenuation capability of these aggregates. Iron slag in the form of micro and nano sizes is utilized to study the influence of particle size on radiation shielding ability. A NaI scintillation detector was utilized to measure the shielding characteristics for the produced mortars at various photon energies ranging from 59.53 keV to 1408 keV. The acquired SEM images of the prepared mortars revealed that addition of nanoparticles enhanced the morphological properties more than the addition of microparticles. The findings demonstrate that the experimental values of the MACs for CM-Ctrl and CM-MIS mortars are in good agreement with those obtained theoretically from the XCOM program. The results revealed that the replacement of waste marble with iron slag increased the mortar density and, in turn, improved the radiation shielding ability. The results also verified that the particle size of the iron slag played an essential role in the shielding efficiency of the mortar. The mortars incorporated by nano iron slag (CM-NIS) had better γ-ray shielding capability than those reinforced with micro iron slag. The importance of the current study is to develop the walls of radiation facilities with low-cost mortars friendly to the environment and capable of absorbing gamma- or X-rays more efficiently. Therefore, studying the radiation shielding features of different waste materials in future work is recommended.

## Figures and Tables

**Table 1:** EDX analysis for cement, waste marble, and iron slag.

Element	Chemical Composition (wt.%)		
	Cement	Waste Marble	Iron Slag
C		11.54	2.53
O	36.45	53.15	39.57
Mg	1.51	0.21	1.39
Al	2.38	0.36	1.59
Si	9.86	0.70	5.47
P	-	-	0.08
S	1.94	-	0.07
Cl	-	-	0.53
K		-	0.07
Ca	45.13	34.04	17.03
Ti	-	-	0.20
Mn	-	-	0.74
Fe	2.73	-	30.73

**Table 2:** The density and the mixing ratios of the mortar samples.

Sample Codes	Composition					Density (g/cm^3^)
	Cement	Aggregate			Water	
		Waste Marble	Micro-IS	Nano-IS		
CM-Ctrl	1	3	-	-	0.5	2.109
CM-MIS	1	2	1	-		2.597
CM-NIS	1	2	-	1		2.635

**Table 3:** Standard radioactive point source energies.

Source	Photon Energy (keV)
Am-241	59.53
Ba-133	80.99
	356.01
Cs-137	661.66
Co-60	1173.23
	1332.50
Eu-152	121.78
	244.69
	778.90
	964.13
	1408.01

**Table 4:** The values of LACs of the tested mortars in addition to lead.

Photon Energy (keV)	Linear Attenuation Coefficient *μ* (cm^−1^)				
	CM-Ctrl	CM-MIS	CM-NIS	Ordinary Mortar [7]	Lead
59.53	0.5972	0.9351	1.1244	0.5158	52.45
80.99	0.4244	0.5848	0.7024	0.3849	23.19
121.78	0.3182	0.4219	0.5052	0.3099	36.60
244.69	0.2394	0.2955	0.3519	0.2407	6.60
356.01	0.2132	0.2609	0.3088	0.2092	3.011
661.66	0.1599	0.1972	0.2319	0.1617	1.173
778.90	0.1525	0.1864	0.2196	0.1502	0.9801
964.13	0.1375	0.1651	0.1927	0.1358	0.7968
1173.23	0.1217	0.1546	0.1783	0.1232	0.6755
1332.50	0.1175	0.1391	0.1601	0.1155	0.6172
1408.01	0.1112	0.1368	0.1566	0.1123	0.5971

**Table 5:** The relative rate of increase in the LACs of CM-NIS compared to CM-Ctrl and ordinary mortar (OM).

E (keV)	(*μ*_CM-NIS_ − *μ*_CM-Ctrl_)/*μ*_CM-Ctrl_ %	(*μ*_CM-NIS_ − *μ*_OM_)/*μ*_OM_ %
59.53	88.28	117.991
80.99	65.50	82.489
121.78	58.77	63.020
244.69	46.99	46.199
356.01	44.84	47.610
661.66	45.03	43.414
778.90	44.00	46.205
964.13	40.15	41.900
1173.23	46.51	44.724
1332.50	36.26	38.615
1408.01	40.83	39.448

**Table 6:** The experimental and theoretical values of MACs and their relative deviations for CM-Ctrl, CM-MIS, and CM-NIS mortar samples.

E (keV)	Mass Attenuation Coefficient (cm^2^/g)						
	CM-Ctrl			CM-MIS			CM-NIS
	Exp.	XCOM	Δ %	Exp.	XCOM	Δ %	Exp.
59.53	0.2832	0.2864	−1.13%	0.3601	0.3648	−1.30%	0.4267
80.99	0.2012	0.1997	0.77%	0.2252	0.2294	−1.84%	0.2666
121.78	0.1509	0.1524	−1.00%	0.1625	0.1601	1.47%	0.1917
244.69	0.1135	0.1152	−1.46%	0.1138	0.1155	−1.48%	0.1335
356.01	0.1011	0.0999	1.22%	0.1005	0.0996	0.88%	0.1172
661.66	0.0758	0.0771	−1.62%	0.0759	0.0767	−0.99%	0.088
778.9	0.0723	0.0716	1.03%	0.0718	0.0712	0.81%	0.0833
964.13	0.0652	0.0647	0.77%	0.0636	0.0644	−1.22%	0.0731
1173.23	0.0577	0.0587	−1.73%	0.0595	0.0584	1.94%	0.0677
1332.50	0.0557	0.0550	1.22%	0.0536	0.0548	−2.17%	0.0608
1408.01	0.0527	0.0535	−1.46%	0.0527	0.0532	−1.04%	0.0594

**Table 7:** The values of HVL, TVL, and MFP of the tested mortars in addition to lead.

E (keV)	CM-Ctrl			CM-MIS			CM-NIS			Lead
	HVL (cm)	TVL (cm)	MFP (cm)	HVL (cm)	TVL (cm)	MFP (cm)	HVL (cm)	TVL (cm)	MFP (cm)	HVL (cm)
59.53	1.161	3.856	1.674	0.741	2.462	1.069	0.616	2.048	0.889	0.013
80.99	1.633	5.426	2.356	1.185	3.937	1.710	0.987	3.278	1.424	0.030
121.78	2.178	7.236	3.143	1.643	5.458	2.370	1.372	4.558	1.979	0.019
244.69	2.895	9.618	4.177	2.346	7.792	3.384	1.970	6.543	2.842	0.105
356.01	3.251	10.800	4.690	2.657	8.826	3.833	2.245	7.457	3.238	0.230
661.66	4.335	14.400	6.254	3.515	11.676	5.071	2.989	9.929	4.312	0.591
778.90	4.545	15.099	6.557	3.719	12.353	5.365	3.156	10.485	4.554	0.707
964.13	5.041	16.746	7.273	4.198	13.947	6.057	3.597	11.949	5.189	0.870
1173.23	5.696	18.920	8.217	4.483	14.894	6.468	3.888	12.914	5.609	1.026
1332.50	5.899	19.596	8.511	4.983	16.553	7.189	4.329	14.382	6.246	1.123
1408.01	6.233	20.707	8.993	5.067	16.832	7.310	4.426	14.704	6.386	1.161

**Table 8:** The values of ratio of HVL for NIS sample and lead.

E (keV)	HVL_CM-NIS_ (cm)	HVL_Lead_ (cm)	Effective Mortar Thickness Equivalent to 1 mm Lead
59.53	0.616	0.013	46.65
80.99	0.987	0.030	33.02
121.78	1.372	0.019	72.46
244.69	1.970	0.105	18.77
356.01	2.245	0.230	9.75
661.66	2.989	0.591	5.06
778.90	3.156	0.707	4.46
964.13	3.597	0.870	4.14
1173.23	3.888	1.026	3.79
1332.50	4.329	1.123	3.86
1408.01	4.426	1.161	3.81

## Data Availability

All data are available in the manuscript.
